# Induced Premature Labour in Certain Diseases of the Mother Not Obstructing Delivery

**Published:** 1896-06

**Authors:** J. G. Swayne

**Affiliations:** Emeritus Professor of Midwifery, University College, Bristol; Consulting Physician-Accoucheur, Bristol General Hospital


### INDUCED PREMATURE LABOUR IN CERTAIN DISEASES OF THE MOTHER NOT OBSTRUCTING DELIVERY.

BY

J. G. Swayne, M.D. Lond.,

*Emeritus Professor of Midwifery, University College, Bristol; Consulting Physician-Accoucheur, Bristol General Hospital.*

(Continued from page 9.)

The next to obstinate vomiting as regards frequency and danger to both mother and child is albuminuria arising from imperfect action of the kidneys, and sometimes complicated by a failure of other excretory organs. Of this kind I have met with four instances. The most dangerous proved to be one in which albuminuria was complicated with jaundice, for in this case both mother and child perished. In the three others all the mothers did well, but two out of the three children died. Before describing these cases I may quote some remarks made by Dr. Barnes, which have a very important bearing on their cause. He says: "At the outset of the discussion we must recognise the fact that the struggle is at first strictly physiological. Let us, then, in the first place, observe the phenomena evoked by pregnancy. The most remarkable are the marvellous exaltation of nervous and vascular tension, and attending alterations in the qualities and quantity of the blood. From the moment that the embryo takes possession of the woman every drop of blood, every fibre, every organ is affected, the entire system is working under high pressure. It is our business to watch narrowly to see how far she is able to bear the strain and to be prepared with means to help her in preserving or in restoring the just equilibrium. Failing in that we endeavour to relieve the overstrained organism by removing the cause of the disturbance, that is to remove the embryo by the induction of labour." [1]

[1] *Brit. M. J.*, 1894, ii. 1419.

These remarks by Dr. Barnes are singularly applicable to the cases I am now about to describe. I will begin with that of albuminuria without other complications.

Case 6.—On May 8th, 1867, Dr. Brittan requested my advice respecting Mrs. F., living in Clifton, who was in the eighth month of her first pregnancy. She was suffering from anasarca arising from albuminuria, for her urine when analysed contained a large amount of albumen. She had also experienced some symptoms of cerebral disturbance, which we considered premonitory of puerperal convulsions. We could not detect any fœtal movements or heart sounds. After consulting we determined to induce labour on the day following. Accordingly, at 10 a.m. on May 9th, I introduced through the speculum a sea-tangle tent into the os uteri, and then plugged the vagina. I removed the tent about 9 p.m. the same evening, and passed a sponge tent of larger size and gave ʒi. of ext. ergot. liquid. in three doses at intervals of an hour. On calling in the afternoon of the next day I found that the os uteri was dilated enough to admit the forefinger, and that some liquor amnii had escaped, but there were as yet no labour pains. Labour, however, came on during the morning of the following day, May 11th, and she soon gave birth to a living female child of fully seven months development. The albuminuria soon began to lessen after the labour, but there was, for several days, some degree of dyspnœa from hydrothorax. The child did very well, and so did the mother ultimately. Thus terminated this most satisfactory case, after an induced labour lasting about forty-eight hours.

The next case, however, is widely different, and not uncomplicated.

Case 7.—On February 11th, 1873, I induced premature labour in Mrs. G., a patient of Dr. Marshall's living in Clifton. He consulted me about her because she had borne only one healthy child at the full term, and five others prematurely, the placenta being always diseased and unusually large. Her health had suffered much during pregnancy, and she had once been jaundiced. When I saw her she was about seven months pregnant. Her urine contained much bile, and occasionally albumen. The uterus was very large and pressed up very much on the stomach and liver. No fœtal heart could be heard, and she had felt no fœtal movements for some days. A slight placental *bruit* was just audible on the left side. Her health seemed so seriously affected by the pregnancy that we determined to bring on premature labour, both on her account and also to save the child, which was evidently becoming very feeble. I therefore introduced a sponge tent on February 9th, 1873, at 10 a.m. This one was removed and a larger one introduced at 10 p.m. There had been slight pains in the interval. The second sponge tent was removed on Monday, the 10th, and a third and larger one passed, which was removed on the morning of Tuesday, the 11th. Slight cutting pains had been experienced for the last two days, and to induce stronger ones ʒi. of ergot was given in two doses. On the evening of that day strong pains had set in, the membranes had ruptured, and the head presented. It was a brow presentation, for the right frontal bone presented with the face towards the right acetabulum. The head felt soft and puffy. By putting my finger into the mouth I brought down the face under the pubic arch. Notwithstanding this the head remained a long time nearly touching the perineum. The pains were feeble, and her general condition rather prostrate.

After a considerable interval the child was expelled. It was universally puffy and œdematous, and the abdomen was distended with ascitic fluid. There was slight pulsation in the cord, but no respiration could be induced. After waiting about half an hour, and finding that the placenta would not come away, I introduced my hand, and found the uterus in a state of hour-glass contraction. The placenta, which was soft and spongy, appeared to be of unusual size, and filled the entire cavity of the uterus above the constriction. In order to detach it I had to pass my hand quite up under the ribs. I found one portion firmly adherent, so that I had to separate it with my nails. I did this with some difficulty, and at last brought away the entire mass, which was found to weigh 2¾lbs. It appeared œdematous, and was of a light buff colour, like one undergoing fatty degeneration. The detachment of the placenta occasioned very little hemorrhage. After delivery Mrs. G. remained in a state of great prostration. The pulse had risen to 140, and much nausea set in. The urine also contained much bile and albumen. She remained in the same low condition, with no sign of rallying, until Friday, February 14th, when she gradually sank, exactly three days after the confinement.

On reviewing the history of this strange and singular case, I cannot but regard it as a very forcible example of the propriety of closely watching Nature under such circumstances, and closely observing how she acts. As Dr. Barnes remarks, "*Natura ostendit viam*," and then he adds: "When seeking to determine the indications for bringing pregnancy to an end, the first source of light will rightly be sought in the observation of the conditions under which Nature herself seeks relief by adopting this course, and the evils incurred if this mode of escape or relief be missed." These words exactly apply to the case before us—a woman who had borne one healthy child, from some cause or other falls into a morbid and unhealthy condition during five succeeding pregnancies, and Nature expels a morbid child and a morbid and preternaturally enlarged placenta before the child is viable, but in time to save the life of the mother. On the next pregnancy, however, Nature is, for some reason, less active in her efforts to get rid of the offending cause, and allows the child to develop up to the age of viability, with the result that both mother and child are lost—the former through extreme exhaustion, aggravated by a most unfavourable labour. Should I ever be consulted in a similar case again, I certainly should advise inducing premature labour before the uterus has risen above the umbilicus, leaving the viability of a probably diseased child altogether out of the question.

Case No. 8.—On September 22nd, 1873, Mr. H. requested me to see his wife with a view to the induction of premature labour. He was a medical practitioner, and told me that his wife, whom I had never seen before, was a multipara, and had in a former pregnancy albuminuria, followed by severe convulsions, so that she nearly lost her life. On this occasion the albuminuria had lasted many days. I found that the albuminous deposit in the test tube formed about four-fifths of the whole quantity of urine. Her face, and also her legs, were very œdematous. She had just completed her eighth month of pregnancy. As it was a clear case for inducing labour without delay, I passed, on September 22nd, 1873, a large tangle tent into the os uteri. This I removed next morning, and was able to insert a large sponge tent. On the next morning (September 24th) I found that she had had several pains during the night. I removed the tent and found that the os was dilated to about the size of a florin, and that the head was presenting. I then punctured the membranes with a stiletted catheter, and drew off a considerable amount of liquor amnii. Labour then came on vigorously, and about 4.45 p.m. she gave birth to a living female child. It cried vigorously at first, but soon became feeble and cold, and died about six hours afterwards. The albuminuria gradually disappeared in a few days after delivery, and the mother made a good recovery.

There is no further comment to make on this case, which would have been a very successful one but for the feeble vitality of the child.

Case 9.—On February 20th, 1877, Mr. Ruddock requested me to see Mrs. I., living at Redland, a primipara who had for some time been under treatment by Mr. Teale, at Leeds, for amaurosis caused by albuminuria. She was very nearly eight months pregnant, and for some weeks had often had as much as 50 per cent. of albumen in the urine. There was also slight dropsy of the lower extremities, with frequent severe pains in the head, and almost total loss of sight. She had not felt the fœtal movements for some weeks, and I could detect no heart sounds or placental *bruit*. On consultation we decided to bring on labour at once. I therefore at 8 p.m., February 20th, proceeded to do so by passing a bougie for about three-quarters of its length between the uterus and membranes, and leaving it there. I saw her the next day at 10.30 a.m. She had passed a good night without pains, but there had been some show, and on withdrawing the bougie I found the os uteri dilated enough to admit the point of the index finger, and I could feel the head covered by the membranes. I then introduced a sponge tent about the size of the index finger. Labour pains set in about an hour after this, and continued until Mr. Ruddock saw her, about 6 p.m. The os was then dilated to the size of a crown, and the membranes presenting. These soon gave way, and the labour ended about 8.30 p.m. The presentation was natural. The child was much decomposed, and had apparently been dead for some weeks. As regards her labour the patient made a good recovery; but in consequence of her leaving Bristol soon afterwards, Mr. Ruddock could give me no information as to any improvement in her vision.

I regret very much that I was not called in to her earlier, so that I might have at once induced premature labour as soon as symptoms of fœtal death were present, as, according to the best authorities in ophthalmic surgery, the induction of labour is the one thing needful to cause improvement of vision in most of these cases, provided there be no organic disease of the kidneys.

I will now relate two cases in which I had to bring on labour on account of great distension of the abdomen, caused by an undue amount of liquor amnii, constituting the diseased condition called hydramnios.

Case 10.—On February 5th, 1875, I was requested to see (in consultation with Mr. Lucy) Mrs. J., living opposite the Clifton College. She was a multipara who had previously had good confinements. By her reckoning she was just seven months pregnant, and I was asked to see her on account of very great œdema of the labia. They were now enormously distended, and so painful that she could scarcely sit down. The legs also were œdematous. On examining the abdomen I found the uterus more distended than it should have been at full term; and I could hear neither fœtal heart nor placenta *bruit*, and could feel no movements. She said she herself had felt none for some days. On making a vaginal examination I could just pass the tip of the finger through the os uteri and feel the tense bag of the membranes. The urine when examined was found to be highly albuminous. There could be no doubt that the case was one of hydramnios, and that the child was probably dead. To relieve the tension of the labia I made several punctures with a fine needle, and a large amount of fluid drained away. We both agreed that it would be advisable to bring on labour. On calling during the next day I found that she had had slight pains after we left, and that the os uteri was dilated to the size of a shilling. I therefore punctured the membranes with a stilette and drew off five pints of fluid. Pains then came on rapidly, and the child was born within an hour. It presented rightly, was a well formed female infant, and weighed 3 lbs. 4 oz. The heart pulsated feebly for a few seconds and then ceased. There was no respiratory effort. After the birth of the child as much more fluid escaped into the bed as had been already collected. The mother did very well.

The next case of this kind was very similar. It was as follows:—

Case 11.—On July 14th, 1883, I induced premature labour in Mrs. K., living in Clifton. She was in her eighth month of pregnancy, and had borne nine children previously. The last of these was born nine years before. I had seen her two days before in consultation with my colleague, Mr. W. Michell Clarke. He then told me that she had symptoms of pregnancy, such as quickening followed by fœtal movements, &c. He had also heard the fœtal heart; but these symptoms had passed off, and latterly she had greatly increased in size. I found that the uterus was large and globular, and of greater size than it would be at full term, and gave a distinct fluctuation. No movements could be felt, or fœtal heart or uterine *souffle* heard. On examining *per vaginam* I could introduce my forefinger through the os uteri and feel the membranes and head of the child. We both regarded it as a case of hydramnios, that the child was probably dead, and that the sooner labour was brought on the better, because the patient was nearly worn out with sleepless nights and pains in various parts of the abdomen. Accordingly, about 10 p.m. July 13th, I introduced an elastic catheter between the uterus and membranes, and left it until 10.0 a.m. the next day. I found then that it had caused slight pain and trifling dilatation of the os. I therefore withdrew it and introduced a full-sized sponge tent. This I left until 3 p.m., when I removed it. The labour pains were then more decided, and the os uteri dilated to the size of a half-crown. I then withdrew the tent and punctured the membranes with Barnes's tent introducer, having previously applied a binder and given ʒi. of ext. ergot. liquid. There was a great gush of liquor amnii, and enough water was expelled to fill a chamber utensil, besides much that was lost. Labour then went on more briskly until the head had come down on the perineum. Here it remained immovable for some time; I therefore applied Simpson's short forceps, and soon delivered her of a still-born female child as large as one would be at full term. The child had evidently been dead for some days, as all the skin had peeled off. Some more ergot was given just before delivery. There was no *post-partum* hemorrhage, and the patient did very well.

In the two cases which have just been described the abnormal condition of pregnancy produced so much distress and inconvenience to the mother, owing to over-distension of the uterus, that it became necessary to deliver without delay. There was no need in either case to wait for the viability of the child, for all signs of its vitality had ceased, so that when it was born it appeared to have been dead for many days; and therefore the mother's life alone was to be considered. It is not quite decided whether the diseased condition producing hydramnios originates with the mother or the child, or perhaps both. For instance, a syphilitic taint may affect both, but more especially the child, and the death of the child is thus a frequent accompaniment of hydramnios. Albuminuria, if present (as in Case 10), or diabetes, would seem to show that a morbid condition of the mother is the primary cause. The greater frequency, however, of hydramnios in twin cases and with female children (as in both my cases) would seem to show that a feeble state of the child is a frequent cause. In my other case (Case 11) the fact that the mother, after bearing many children, bore no more for nine years, until after that time, just before the menopause, she again became pregnant, had hydramnios and gave birth to a dead child, would seem to favour this idea.

The next case I have to relate occurred early in pregnancy, and is rather a case of protracted abortion attended with so much hemorrhage as to endanger the life of the woman, and render it necessary to induce the expulsion of the fœtus and thus cut short the pregnancy.

Case 12.—On August 21st, 1876, I was sent for to see Mrs. L., a multipara, living in Clifton, who was nearly five months pregnant. For five weeks she had almost daily hemorrhage from threatened abortion, which had resisted all treatment and had gradually reduced her strength to the lowest point. The os was slightly open, but the membranes could not be reached. Feeling confident that the child was dead, and fearing any further reduction of the mother's strength, I determined to bring on labour. About 10 p.m. I introduced a medium-sized tangle tent. I was sent for about 6 the next morning, because she had experienced some labour pains and also more hemorrhage. I removed the tent and found the os uteri partly dilated. I then introduced a full-sized tent. I called at 3 p.m. on that afternoon and removed the last tent. The os was well dilated, there were regular pains, and the membranes were protruding. I was again sent for about 5 p.m., and when I arrived I found that both the child and placenta had been expelled. The child, from its appearance, must have been dead for two or three days. The mother made a very good recovery.

The case just related accords with my experience of impending abortion during the first half of utero-gestation, which is, that if pain, hemorrhage, and other symptoms of abortion cannot be checked by extreme quiet, rest, and sedatives during the first week, it is generally useless to persevere with these measures any longer; for Nature is evidently bent on getting rid of the offending cause, which may be either diseased membranes, a partially detached or misplaced placenta, or a dead fœtus. A woman who is kept in the recumbent posture under these circumstances, and allowed to bleed slowly every day for weeks, will ultimately die from loss of blood unless the fœtus be expelled by the natural efforts or by the assistance of Art.

The last case I have to mention is a curious and anomalous one, and also very unusual. It is as follows:

Case 13.—Mrs. M., living at Kingsdown, Bristol, consulted me, on account of intense irritability of the bladder towards the latter part of pregnancy. She was confined at her full time about fourteen months previously to this labour, and suffered a good deal from the same affection towards the end of her pregnancy, but still nothing in comparison to that of the present time. The dysuria was much aggravated by taking food or drink. Sedatives of every kind, both local and general, were tried, but with very little relief, until at last her condition became most critical. She was disturbed every hour in the night to pass water, and the suffering and want of rest thus produced at last caused extreme emaciation, great irritability of the stomach, and even slight symptoms of mental aberration. From her reckoning, which was somewhat doubtful, because she could not be sure of the date of her last menstruation, I considered her to be about seven months pregnant. Although reluctant to induce labour so early, yet I dreaded the results of further delay, and on January 18th, 1875, about mid-day, passed an elastic bougie about eight inches between the uterus and membranes, and, coiling up the end, left it in the vagina. On the next day I saw her at 12, and removed it. There had been slight pains about once every hour since the day before, and the os uteri would just admit the point of the index finger. I then introduced a full-sized sponge tent. I was called up to her during the night. I found that she had had strong pains, and that the os was nearly fully dilated. The membranes were soon ruptured, and the child was born at 3.15 a.m. It was a male, and weighed 3 lbs. 1 oz. and measured 15 inches in length. Although it breathed well and cried strongly, it lived only twenty-four hours. The mother did well.

Dr. Barnes, at the end of his discussion, remarked very justly that "the rule urged by Denman not to undertake the induction of labour or abortion without a formal and deliberate consultation is imperative." Fully agreeing as I do with this remark, I must confess that in these two last cases I acted without a previous consultation, and therefore feel bound to excuse myself for so doing. The fact was, that in neither case did I feel conscious of sacrificing the infant's life in order to save that of the mother. In the first case, I felt certain that the child was already dead, and this was proved by its appearance when born; in the second, I felt sure that the child had become viable, for there had been no previous signs of its death, and its size and apparent vigour when born, especially as regards respiration, made us think that I was right in my opinion as to its viability. But in this I was doomed, after it had lived a short time, to be disappointed. The state of the mother, however, was such that I dared not defer the operation any longer. This was very disappointing, for most probably, had the condition of the mother permitted it, an extra fortnight might have made all the difference in favour of the child.

I have now mentioned all the diseased conditions of the mother which rendered it necessary for her safety (sometimes quite irrespectively of that of the child) that I should cut short the pregnancy.

There are other diseases besides those mentioned—such, for instance, as heart-disease, aneurysm, fibroid or ovarian tumours, phthisis, pernicious anæmia, &c.—but I have no record of any cases of this kind complicating pregnancy; although they are very rare, they have occurred in the practice of others, and have required artificial delivery.

In the course of my midwifery practice I have had to induce premature labour in twenty-four cases on account of *pelvic obstruction.* I shall say no more about these now, except to speak of the safety of induced labour under such circumstances, compared with that under such as I have already related. As regards the mother, there is no doubt that the operation is incomparably safer when undertaken on account of pelvic obstruction than when done to obviate the results of disease.

In the first of these two classes—viz., the twenty-four cases operated upon on account of pelvic malformation—there was only one maternal death, and that arose from a very unusual cause; viz., ruptured uterus arising from an arm presentation, in which the child was spontaneously expelled in a doubled-up condition.

In the second class—viz., the thirteen cases I have already related—there were three maternal deaths. The first of these resulted from most obstinate vomiting just before full term, when, in hope that natural labour might occur daily, induction was probably delayed too long. In the second, the placenta was much diseased, and the mother suffering from jaundice and anasarca, as well as albuminuria. In the third, the intense vomiting which destroyed life evidently arose from hard drinking habits, to which the patient had given way not long before she became pregnant.

The causes why there should be such a great difference between these two classes as regards the safety of the mother are very obvious. As Spiegelberg remarks: "The operation is either a prophylactic measure, or else a rapid means of rescue from danger." As I have shown, it is prophylactic when there is mechanical disproportion, as in the first class, and can be performed under the most favourable circumstances; the woman is in her usual health, the operation is deliberately performed and at a carefully chosen time. Hence in my own twenty-four cases there would have been no maternal death, if we exclude the one unusual case. If we admit it however, the mortality would be no more than $4\frac{1}{4}$ per cent.; whereas in the second class of cases the operation, as Spiegelberg remarks, is a means of rapid rescue when the mother is suffering from dangerous disease, and it consequently has to be performed at the most critical moments, when we cannot choose our own time, when she is, perhaps, *in extremis*, and under the most disadvantageous circumstances, both for her and the child. Hence, in my own list of thirteen such cases there were three maternal deaths, or about 24 per cent. mortality. The causes I have just mentioned would also add to the comparative danger of the child, but not to so marked a degree. However, I cannot enter upon this at present. Towards the end of his address, Dr. Barnes spoke very strongly of the danger of high vascular tension "with ultra-physiological changes in the qualities and quantities of the blood. . . . This is demonstrated by quickened pulse and the indications given by the sphygmograph. This pressure is essentially eccentric or towards the periphery." And then he gives instances of external hemorrhages or effusions of serum thus produced, and adds the following excellent remarks as to treatment, which my own experience thoroughly endorses. He says: "Venesection is sometimes strongly indicated, as when hæmorrhages occur, and when there is undue pressure on the kidneys, as in Mahomed's pre-albuminuric stage, or in the early stages of albuminuria with convulsion. Venesection is out of date, but I have practised it with the best results. Then in the last resource we follow Nature's most decisive action in her rebellion against pregnancy by inducing labour." With such testimony as this in its favour we may well ask, Why should venesection be "out of date?" I never could find any good reason why it should be, especially in cases of great arterial tension, and I can quote, on the contrary, a case which conclusively proves that any practitioner is guilty of neglect who in *every* case at once adopts "the last resource," viz., the induction of labour, without first trying so valuable a remedy as venesection. I can quote a case of this kind which occurred to my brother, Mr. S. Henry Swayne, and which was read by him at the meeting of the British Medical Association in Bristol in 1863. It was to this effect: A woman in the seventh month of pregnancy was attacked with puerperal convulsions, for which blisters, cold to the head, purgatives and enemata were administered without effect. The urine, on being examined, contained so large a quantity of albumen that the deposit formed by it occupied about $\frac{5}{6}$ of the whole bulk of the fluid examined. She was bled on the next day to ℥xxv. No more convulsions occurred. Some urine, passed within twenty-four hours after the bleeding, was examined, and the albumen was found to occupy only $\frac{1}{6}$ of the whole volume after subsidence. Although the fœtus appeared to be dead, no attempt was made to deliver, because there was no dilatation of the os uteri. The woman went on well for twelve days, when labour set in and terminated favourably, without convulsions, in the birth of a dead child, somewhat decomposed. My brother remarked: "No other exciting cause for the convulsions in this case beyond the uræmia could be discovered; and that the presence of the fœtus in utero had little share in bringing them on or maintaining them, would seem to be shown by the cessation of the convulsions and of the albuminuria, notwithstanding the presence of a dead child in the uterus . . . for a fortnight after. . . . That the improvement was due to the measures employed, especially to the bleeding, can, I think, scarcely admit of a doubt."[1] With this remark I entirely agree, for it was a most conclusive case. I cannot understand how such cases can be ignored by men who contend, as many do in the present day, that induced labour is the one thing needful in cases of persistent albuminuria, even to the extent of having recourse to the "accouchement forcé"—a mode of practice strongly reprobated by such high authorities as Charpentier and others of the French school. In the discussion which ensued after Dr. Barnes's introduction, Professor Byers of Belfast remarked very truly: "As to the presence of albumen, this in itself should not in all cases be considered an indication for terminating pregnancy." The case I have just quoted fully confirms this opinion.

[1] *Brit. M. J.*, 1863, i. 648.

